# Properties of Naked Silver Clusters with Up to 100 Atoms as Found with Embedded-Atom and Density-Functional Calculations

**DOI:** 10.3390/molecules28073266

**Published:** 2023-04-06

**Authors:** Shivangi Garg, Navjot Kaur, Neetu Goel, Mohammad Molayem, Valeri G. Grigoryan, Michael Springborg

**Affiliations:** 1 Theoretical and Computational Chemistry Group, Department of Chemistry, Centre of Advanced Studies in Chemistry, Panjab University, Chandigarh 160014, India; 2 Department of Chemistry, Faculty of Science, SGT University, Gurugram 122505, India; 3 Physical and Theoretical Chemistry, Department of Chemistry, University of Saarland, 66123 Saarbrücken, Germany; 4 Laboratory of Theoretical Chemistry, Department of Chemistry, Namur Institute of Structured Matter (NISM), University of Namur, Rue de Bruxelles 61, 5000 Namur, Belgium

**Keywords:** Ag clusters, structures, energetic properties, electronic properties, stability, reactivity

## Abstract

The structural and energetic properties of small silver clusters Ag${}_{n}$ with *n* = 2–100 atoms are reported. For *n* = 2–100 the embedded atom model for the calculation of the total energy of a given structure in combination with the basin-hopping search strategy for an unbiased structure optimization has been used to identify the energies and structures of the three energetically lowest-lying isomers. These optimized structures for *n* = 2–11 were subsequently studied further through density-functional-theory calculations. These calculations provide additional information on the electronic properties of the clusters that is lacking in the embedded-atom calculations. Thereby, also quantities related to the catalytic performance of the clusters are studied. The calculated properties in comparison to other available theoretical and experimental data show a good agreement. Previously unidentified magic (i.e., particularly stable) clusters have been found for n>80. In order to obtain a more detailed understanding of the structural properties of the clusters, various descriptors are used. Thereby, the silver clusters are compared to other noble metals and show some similarities to both copper and nickel systems, and also growth patterns have been identified. All vibrational frequencies of all the clusters have been calculated for the first time, and here we focus on the highest and lowest frequencies. Structural effects on the calculated frequencies were considered.

## 1. Introduction

Metal-derived nano-clusters containing two to a few thousands of atoms are of great interest because of their outstanding properties. These properties, which have their roots in the large surface-to-volume ratio combined with having a size below that of the thermodynamic limit make clusters good candidates for building blocks of nano-structures and nano-electronics as well as in catalysis. The bonding in alkali and alkaline earth metals are largely metallic, delocalized, and non-directional primarily due to valence *s* orbitals. On the other hand, *sp*-metals like aluminium show some directional bonding due to the *s* and *p* orbitals. But transition metals possess a bonding with a larger degree of covalency and higher directionality involving also the valence *d* orbitals.

Although coinage metals possess properties resembling those of the transition metals, due to their ($n-1)\;d^{10}$ ns electronic configurations, they also have a half filled *s* orbital so that the share properties with those of the alkali metals. In fact, it is the degree of *s-d* hybridization that can be considered responsible for various of their interesting properties.

Among the coinage metal clusters, silver clusters have received special attentions because of two reasons. First, the spatial overlap of the outer *d* orbitals with the partially filled *s* orbitals makes their study interesting and also challenging. The second reason is the possible applications of silver clusters for optoelectronics and catalysis. In addition to these facts, small silver clusters can exhibit an interesting size dependent gap between the highest occupied and the lowest unoccupied molecular orbitals (HUMO-LUMO gap). Therefor silver clusters have been at the focus of many experimental and theoretical studies.

Experimental studies have provided important information on Ag${}_{n}$ clusters. Thus, by analyzing the frequencies and intensities of the vibrational modes, a planar trapezoidal structure for Ag${}_{5}$ has been suggested, in contradiction to the trigonal-bipyramid with a Jahn-Teller distortion proposed by Howard et al. based on results from Electron Spin Resonance Spectroscopy (ESR) [1,2]. In another study, Xing and co-workers carried out trapped ion-electron diffraction measurements and found that a local order with fivefold symmetry dominates the cluster structures of Ag${}_{n}^{+}$ for n<55 (but n=38 was found to be an exception) [3]. The same method has also led to the suggested icosahedral motifs for the lowest energy isomer of Ag${}_{n}^{+}$, *n* = 19–79 [4]. Photoelectron spectra of Ag${}_{n}^{-}$ (n≤21) with different photon energies, revealed sharper spectral patterns of Ag in comparison to alkali-metal cluster. This has been interpreted as a result of stronger bonding in silver clusters and overlap of the valence *d* and *s* orbitals [5].

Also theoretical methods have been applied to study silver clusters. These can be split into two categories: those that use semiempirical potentials and those that are based on first-principles approaches. Here, the main difference of these two approaches, like in any other many-body problem, is in the limits imposed by system sizes. Both approaches have advantages and disadvantages. Thus, the more approximate methods allow for studying a larger range of also larger clusters, but with the cost of a reduced accuracy. Such studies are, therefore, best suited for identifying general trends and include the study of the present work.

On the other hand, first-principles calculations provide more accurate results but often on only smaller systems and a smaller range of systems and structures. Thus, neutral, cationic, and anionic silver clusters of the sizes *n* = 5–9 have been investigated using a second-order many-body perturbation theory with a Hay-Wadt effective core potential, and it was found that neutral clusters with up to 6 atoms favor planar geometries, but the charged clusters with more than 6 atoms prefer three dimensional (3D) structures [6,7]. Studies based on using ab initio, quantum chemical techniques and core potentials (in the size range *n* = 2–9) confirm the tendency of silver clusters larger than tetramers to adapt 3D geometries, and suggest a competition between stability of 2D and 3D structures that is more pronounced for neutral clusters than for cationic ones [8,9,10]. Static polarizabilities and optical absorption spectra of Ag${}_{n}$ clusters with *n* = 2–8 were investigated using a time-dependent density functional method (DFT) [11]. The static polarizabilities show even-odd oscillations for n≤6, while for n≥7 these decrease. This can be interpreted as a result of structural transitions from 2D to 3D at n=7. Gradient-corrected DFT calculations found that silver clusters in the range *n* = 9–20 have a transition from double-layered platelike structures, favored for *n* = 9–16, to spherical compact configurations for n≥17 [12].

Also more phenomenological approaches have been applied to silver clusters. Thus, Michaelian et al. used a genetic algorithm (GA) to search the potential energy surface (PES) as obtained using an *n*-body Gupta potential of some selected sizes (*n* = 6, 7, 12, 13, 14, 19, 55) [13]. They found icosahedral structures for the energetically lowest isomers of all their selected Ag clusters except for the cases n=38 and 75 where fcc and decahedral structures, respectively, were found. A tight-binding model together with a GA search method was applied to explore the PES of silver clusters with up to 21 atoms by Zhao et al. [14]. The results show that an icosahedral growth pattern starts and dominates for Ag${}_{n}$ with n≥11. Magic clusters were found for n=2,8,14,18,20 which all have a closed electronic shell. The random tunneling algorithm as a global optimization method, together with the Gupta and Sutton-Chen (SC) potentials were used by Shao et al. to investigate silver clusters in the range *n* = 3–80 [15]. Different growth patterns were identified in the *n* = 15–47 range for these two different potential models. While structures obtained using the Gupta model showed a tendency towards disordered motifs, the SC results seemed to favor more ordered structures. Different versions of the Gupta potential combined with an Aufbau/Abbau search strategy was employed in an earlier work by some of us to study Ag${}_{n}$ for 2≤n≤150 [16]. Thereby, two types of the embedded atom method (EAM) were used to study clusters with *n* up to 60. The many-body potentials of Rosato, Guillopé, and Legrand (RGL) in combination with a MD simulation of silver clusters with *n* up to 150 gave that at smaller sizes (n<100) motifs based on icosahedra and Marks decahedra are found as the minimum-energy structures [17]. However, for 100≤n≤150, the growth conditions have significant impacts on the results and different magic structures are found as minimum-energy clusters.

For a deeper understanding of the properties of the clusters, knowledge of their vibrational frequencies is needed. Among others, these frequencies can be used to determine the thermodynamics of the clusters and their finite-temperature properties. They can also provide information on scattering and kinetic effects in the clusters. It is furthermore possible to study structures and bonding between atoms using the vibrational frequencies. In spite of this importance, up to now, the vibrational frequencies of a wide range of fully optimized silver clusters have not been determined, neither theoretically nor experimentally. Just for few, small and selected sizes DFT methods have been employed to determine these frequencies [18].

Although many studies have focused on silver clusters and their properties a thorough, unbiased search for structures of global total-energy minima is lacking for a wider range of cluster sizes. Based on our knowledge, up to now, no study has been carried out for pure silver clusters using a more precise potential model like the EAM in conjunction with an unbiased global optimization method to investigate a wider range of cluster sizes beyond *n* = 2–20. In this paper we describe the results of an intensive search for the lowest energy isomers of Ag${}_{n}$ with *n* = 2–100. Using the EAM as the model potential for interatomic interactions we applied the basin-hopping global optimization method, developed by Wales, to identify the global minimum structures on the PES [19,20,21]. Subsequently, we use the optimized structures for *n* = 2–11 as input for additional accurate DFT (density-functional-theory) calculations. Thereby we obtain useful information on the electronic properties of those clusters that also can be generalized to the larger ones. Moreover, these calculations will also allow for estimating the accuracy of the EAM calculations.

Our paper is organized as follows. In Section 2, we describe the total-energy models and the global optimization method. The results and discussions for energetic, electronic, and structural properties are presented in Section 3. At the end, the conclusion and a summary are the content of Section 4.

## 2. Computational Methods

### 2.1. The Embedded-Atom Method

Although the EAM was developed to study metallic bulk systems, previous studies on clusters based on this method have shown that it can model the interactions in metallic clusters satisfactorily [16,22,23,24,25,26,27]. To model the interatomic interactions of silver clusters we have used the version of the EAM developed by Daw, Baskes, and Foiles [28,29,30,31]. The main concept of the EAM is to treat each atom of the system as an impurity that is embedded into the host provided by all other atoms of the system. The energy required to embed each atom into the host is a functional of the local electron density formed by the host atoms at the position of the embedded atom. In addition, we add pair interactions between all atoms. Hence, the total energy of a metallic system is written as [30,31]

(1) $$E_{tot}=\sum_{i}F_{i}\left(\rho_{i}^{h}\right)+\frac{1}{2}\sum_{i,j(i\neq j)}\phi \left(R_{ij}\right).$$

Here, $F_{i}\left(\rho_{i}^{h}\right)$ is the embedding energy, i.e., the energy required to embed atom *i* into the host of all other atoms. $\rho_{i}^{h}$ is the local electron density at site *i* from all other atoms. $\phi_{ij}\left(R_{ij}\right)$ is a short-range repulsive interaction between atoms *i* and *j* which are separated by a distance of $R_{ij}$. $\phi_{ij}\left(R_{ij}\right)$ is written as

(2) $$\phi_{ij}\left(R_{ij}\right)=\frac{Z_{i}\left(R_{ij}\right)Z_{j}\left(R_{ij}\right)}{R_{ij}},$$

where $Z_{i}\left(R_{ij}\right)$ and $Z_{j}\left(R_{ij}\right)$ are effective charges of atom *i* and *j*. The local electron density of the host atoms at the position of atom *i* is written as a superposition of their atomic electron densities [30,31],

(3) $$\rho_{i}^{h}=\sum_{j(\neq i)}\rho_{i}^{a}\left(R_{ij}\right).$$

where $\rho_{i}^{a}\left(R_{ij}\right)$ is an averaged electron density of atom *j* at the position of atom *i*.

The atomic electron densities are determined by means of Hartree-Fock calculations [32,33]. To define $F_{i}$ and $\phi_{ij}$, their adjustable parameters have been set by fitting to bulk properties of the metal like lattice constant and sublimation energy [29,30,34].

### 2.2. The Basin-Hopping Method

The major problem of any unbiased global optimization study is the numerous minima on the PES. The number of these minima increases exponentially with the system size [20]. Different methods for tackling this problem have been presented, including genetic algorithms [35,36], simulated annealing [37], undersurface deformation methods, and quantum tunneling methods [38,39]. The basin-hopping method (BH) which we have chosen as our search strategy is based on undersurface transformation, and was originally developed by Wales et al. [19]. The BH has been successfully applied to locate all global minima of pure and binary Lennard-Jones clusters with sizes up to 110 and 100 atoms, respectively [19,40].

With this method, the PES can be mapped onto a staircase topography. This mapping is given as

(4) $$\tilde{E}\left(\vec{X}\right)=\min\left\{E\left(\vec{X}\right)\right\},$$

where $\tilde{E}$ is the transformed energy, $\vec{X}$ represents the 3n-dimensional vector of all nuclear coordinates, and the min emphasizes a local minimization starting from $\vec{X}$. Subsequently, the transformed energy landscape, $\tilde{E}$, is treated within a Monte Carlo (MC) algorithm at a constant temperature *T*. In each step of the MC simulation, the structure is gradually changed and a new one is accepted if it is lower in energy with respect to the previous lowest-energy structure. If not, it survives with a probability of $\exp[\frac{\left(E_{\mathrm{old}}-E_{\mathrm{new}}\right)}{k_{B}T}]$, ($k_{B}$ is the Boltzmann constant and $E_{\mathrm{old}}$ and $E_{\mathrm{new}}$ are the so far lowest total energy and the new total energy). Using this probability, the system hops with a thermal energy of $k_{B}T$ from one basin to another. This hopping helps to avoid that the calculation is trapped in local minima [20,21].

### 2.3. DFT Calculations

Starting with the geometries for Ag${}_{n}$, *n* = 2–11, we performed density functional calculations without imposing any symmetry restrictions and using the Gaussian 09 program package [41]. We used the BPV86 density functional based on Perdew’s 1986 functional together with the local correlation functional by Vosko et al. (VWN) [42] in conjunction with the LanL2DZ (Los Alamos National Laboratory 2 Double-Zeta) basis set [43]. The LanL2DZ basis set has been extensively shown to provide valuable results of a series of bimetallic clusters [44,45,46]. Vibrational frequencies and natural bond orbital (NBO) analysis were computed at the same level of theory [47]. In some cases, imaginary vibrational frequencies were found, whereby the symmetry of the cluster was reduced in order to obtain stable systems.

## 3. Results and Discussion

### 3.1. Structural Properties

At first, we check the validity of our EAM approach that has been parameterized for bulk properties by comparing the calculated dimer bond length to experimental values. The EAM finds 2.4433 Å which agrees well with the experimental value of 2.53 Å [48,49]. For the DFT approach, we find a bond length of 2.58 Å. The vibrational frequency for the dimer is found to be 225.7 cm${}^{-1}$ in the EAM calculations, compared with 188.9 cm${}^{-1}$ in the DFT calculations and an experimental value of 192.4 cm${}^{-1}$ [50]. Again, a good agreement is found.

**Figure 1:**
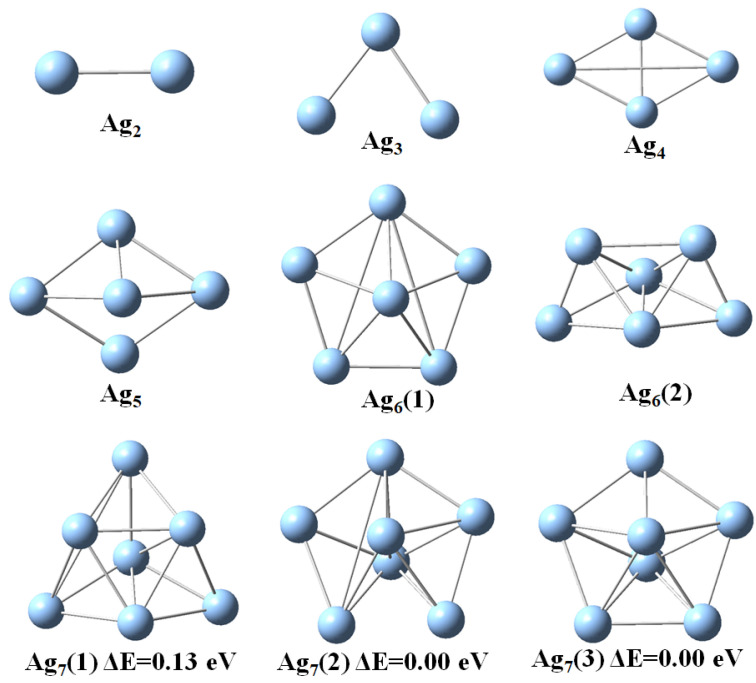
Optimized geometries of of Ag${}_{n}$ (*n* = 2–7) clusters as obtained with the DFT calculations. The labels refer to the energetic ordering according to the EAM calculations.

**Figure 2:**
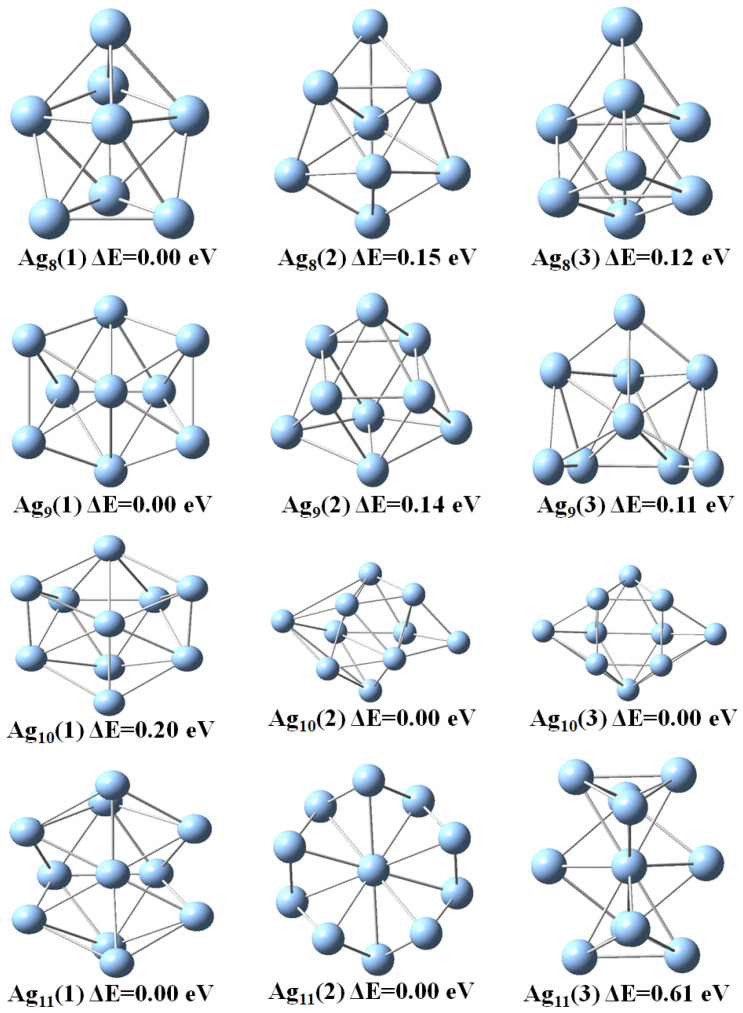
Optimized geometries of of Ag${}_{n}$ (*n* = 8–11) clusters as obtained with the DFT calculations. The labels refer to the energetic ordering according to the EAM calculations.

Next, we discuss the results from the DFT calculations in some detail. The optimized geometric structures of stable Ag${}_{n}$ (*n* = 2–11) clusters as obtained with the DFT calculations are represented in Figure 1 and Figure 2 and the point symmetries are listed in Table 1. In order to faciliate the comparision with the results of the EAM calculations we have used labels according to the energetic ordering from the EAM study. If the cluster size is even, the ground state is singlet, and doublet otherwise. Thus, we see that the energetic ordering changes for many cluster sizes so that even the most stable clusters of the EAM study is not always the most stable clusters of the DFT study. In two cases, Ag${}_{7}$ and Ag${}_{10}$, two different structures of the EAM study become identical according to the DFT calculations. That two different computational methods yield different energetic ordering of clusters is not uncommon and will most likely also be the case for larger Ag${}_{n}$ clusters.

Next, we focus on the most stable clusters according to the DFT calculations. For these, the Ag${}_{3}$ and Ag${}_{4}$ clusters have planar structures with $C_{2v}$ and $D_{2h}$ point group symmetry with average bond lengths of 2.65 and 2.77 Å [8]. The $C_{2v}$ distorted trigonal bipyramid geometry and pentagonal pyramidal with $C_{5v}$ point group symmetry was found for the stable Ag${}_{5}$ and Ag${}_{6}$ clusters, respectively. Thus, in these calculations the transition from 2D to 3D structures occurs for n=5. For *n* = 7 to 11, Ag${}_{n}$ clusters have three different structural isomers. The most stable heptamer of Ag has a 3D structure of $D_{5h}$ symmetry which is consistent with a previous study [10]. The Ag${}_{8}$ clusters with $C_{s}$ and $C_{2v}$ symmetry are 0.15 and 0.12 eV less stable than $D_{2d}$ symmetric structure, respectively. Two other isomers of Ag${}_{9}$ cluster with $C_{1}$ and $C_{s}$ symmetry are 0.14 and 0.11 eV less stable than the $C_{2v}$ structural isomer, respectively. The global minima for the Ag${}_{10}$ and Ag${}_{11}$ clusters also adopt the $C_{2v}$ symmetry.

Next we discuss the symmetry properties of the larger Ag${}_{n}$ clusters as found with the EAM calculations in some more detail. These calculations resulted in the symmetry point groups of the three lowest lying isomers of each size as listed in Table 2. We see that most clusters have fairly low symmetries. Moreover, high symmetries are often occurring for the energetically lowest isomer although there are also several exceptions to this rule (for instance, n=18). The subsequent DFT calculations on the clusters with *n* up to 11 did in many cases not change the symmetry properties, although in some few cases the symmetry was reduced, but only in very few cases, a structure of higher symmetry resulted; see Table 1. The lowering of the symmetry can be explained through electronic effects like Jahn-Teller distortions that are not included in the EAM calculations.

There are only few experimental results that these can be compared with. Trapped ion electron diffraction experiments at a temperature of 100 K showed that Ag${}_{19}^{+}$ favors icosahedral structures. The energetically lowest isomer and the third isomer for this cluster size were observed to possess $C_{s}$ and $D_{5h}$ symmetries, respectively, while EAM-BH finds $D_{5h},C_{1}$, and $C_{s}$ as point groups of 19.1,19.2, and 19.3, respectively [4]. Also, the obtained icosahedral structure ($I_{h}$) for Ag${}_{55}$ is in agreement with experimental data for both cationic and anionic silver clusters [4,51]. The differences in ordering of structures with different symmetries, can be the result of the extra charge in the cationic and anionic clusters, studied in experiments, and also temperature effects but certainly also inaccuracies in the EAM. In our EAM study, we have considered ground state case which corresponds to zero temperature.

The present results agree well with those found with the DBF version of the EAM and with the *n*-body Gupta potential [16] with only few exceptions. These are related to the differences in the potentials but could also be related to differences in the methods applied for searching for the total-energy minima. In order to understand the consequences of the latter, we shall here compare the symmetries of the Ag clusters obtained using two different minima searching methods, i.e., the BH here and the variable metric/quasi-Newton method combined with our own Aufbau/Abbau algorithm of our earlier work [16].

For high-symmetry cases like Ag${}_{13}$, Ag${}_{19}$, Ag${}_{28}$, Ag${}_{55}$, and Ag${}_{75}$ both approaches find structures with the same symmetries of all three lowest energy isomers, Table 2. But the $D_{3h},D_{3h}$, and $C_{2}$ symmetries are found for 23.1,23.2, and 23.3 with the BH method, whereas the Aufbau/Abbau method leads to $D_{3h},C_{2}$, and $C_{s}$, respectively. The energy difference for 23.1 and 23.2, both with $D_{3h}$ symmetry according to EAM-BH, is about 0.082 eV, i.e., small. The 23.1 isomer has a more compact shape whereas the 23.2 is more oblate. For Ag${}_{38}$ only the symmetries of the third isomer differ. Basin-hopping finds $C_{s}$ while from Aufbau/Abbau it is a $C_{5h}$ structure.

Our results for structures for n=6,7,13,14,19,38,55 and 75 agree well with those found by Michaelian et al. in their *n*-body Gupta study [13]. In another study, using the Sutton-Chen 12-6 potential for the interatomic interactions, all symmetries of their lowest isomer structures for n≤22 are recovered in our calculations here [52].

The three energetically lowest isomers of the present study on Ag clusters and those of nickel (Ni) and copper (Cu) clusters, all calculated with the same version of the EAM but using different global optimization methods, have some similarities in regards to the symmetry point groups but also differences [53,54]. For the lowest energy isomer of clusters with particular stability, i.e., n=13,19,23,28,38,55, and 75, all the three metals have the same symmetries. For the second and third isomers, however, some differences appear. Whereas the Ag${}_{13}$ and Ni${}_{13}$ clusters have the $C_{s}$ symmetry for both their second and third isomers, Cu${}_{13}$ possesses the more symmetric structures of $D_{5h}$ and $O_{h}$, respectively. At n=19, $C_{1}$ is the point group of the second isomer of all three metal clusters, but the 19.3 of Cu has a $C_{1}$ rather than a $C_{s}$ structure, which is also the case for Ag and Ni. Although all 38.1 structures of these metals are of $O_{h}$ symmetry, the second isomers of Ni${}_{13}$ and Ag${}_{13}$ are structures with $C_{5v}$ symmetry and for the Cu${}_{13}$ cluster, a $C_{5}$ structure is obtained. The point groups for the 38.3 clusters of all three metals are different, $C_{s},C_{5v}$, and $C_{5}$ for Ag${}_{38}$, Ni${}_{38}$, and Cu${}_{38}$, respectively. For n=55, both the 55.1 and 55.2 isomers of these metal clusters have the same symmetries. This is also the case for n=75. Finally, for n≥80 common symmetries are observed more frequently for Ag and Ni than for Ag and Cu.

A more detailed description of the structural similarities between different types of clusters can be obtained with the help of the concept of similarity functions [53]. The similarity function for the comparison of two structures with the same number of atoms is defined according to

(5) $$S=\frac{1}{1+q/u_{l}}$$

with $u_{l}$ being a scaling constant (below set equal to 1). In this equation, *q* is given through

(6) $$q={\left[\frac{1}{n_{t}}\sum_{i=1}^{n_{t}}{\left(s_{i}-s_{i}^{\prime }\right)}^{2}\right]}^{1/2}$$

with $n_{t}$ being the number of terms in the summation. Moreover, for each of the two clusters (marked with unprimed and primed symbols, respectively) we define the radial distances of each atom in each cluster with respect to the center of the cluster,

(7) $$r_{i}=\left|\vec{R_{i}}-\vec{R_{0}}\right|.$$

where $\vec{R_{0}}=\frac{1}{n}\sum_{j=1}^{n}\vec{R_{j}}$ is the cluster center. Alternatively, we consider all interatomic distances. The quantities $s_{i}$ and $s_{i}^{\prime }$ are then either of these two possibilities: the radial distances of the interatomic distances. Subsequently, the two sets of distances, $\{s_{i}\}$ and $\{s_{i}^{\prime }\}$, separately are sorted in increasing order and scaled with the lattice constants of the crystalline system in order to take differences in bond lengths into account. These scaled distances are used in Equation (6). For similar structures, *S* approaches 1 and for very different structures it will approach zero. Our experience suggests that a threshold value around 0.7 separates similar and dissimilar structures.

**Figure 3:**
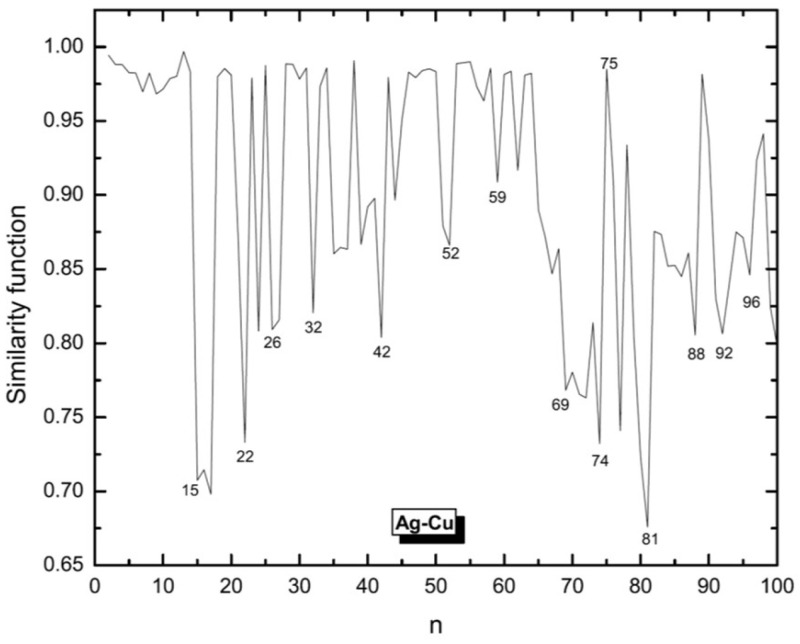
Similarity function for the comparison of silver clusters with copper clusters. Structures of both systems have been defined by the same EAM potential model but global optimization of Ag clusters have been done by BH and those for Cu are the results of Aufbau/Abbau optimizations. The similarity function is based on the radial distances.

**Figure 4:**
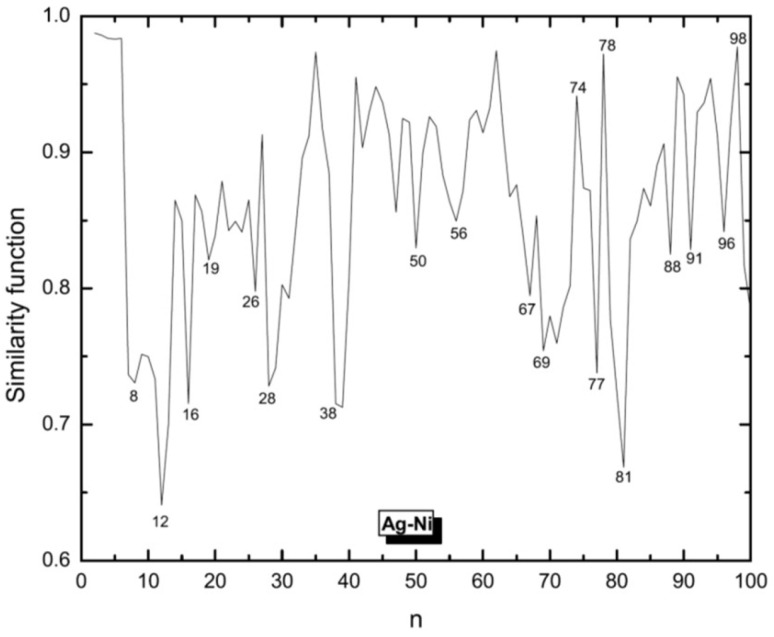
Similarity function for the comparison of silver clusters with nickel clusters. Structures of both systems have been defined by the same EAM potential model but global optimization of Ag clusters have been done by BH and those for Ni are the results of Aufbau/Abbau optimizations. The similarity function is based on the radial distances.

We use this function to compare Ag clusters with Cu and Ni clusters for which the structures were obtained in previous studies [53,54]. The similarity functions for the comparison of Ag clusters with Cu and Ni clusters are shown in Figure 3 and Figure 4, respectively.

In general, Ag clusters appear as being more similar to Cu rather than to Ni clusters. For smaller sizes, Ag and Cu show higher values of similarity and those for Ag and Ni are smaller. But with increasing cluster size, also Ni clusters become more similar to Ag clusters, although still less similar as Cu and Ag. But for n>81, the similarity function suggests that the Ni clusters then are the ones being most similar to Ag clusters.

**Figure 5:**
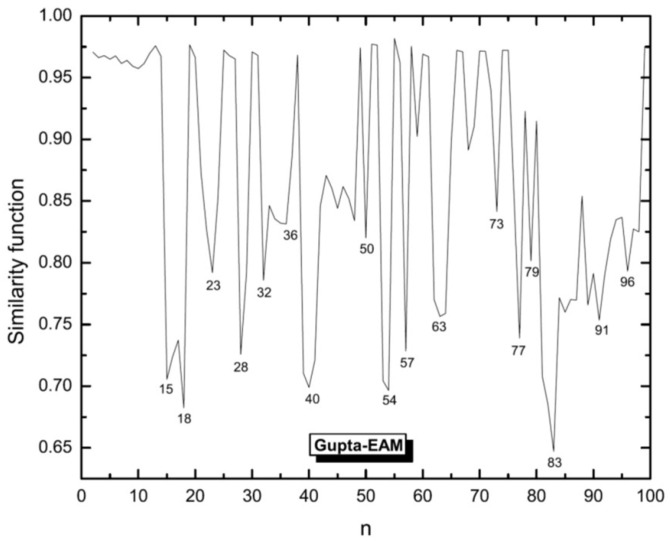
Similarity function for silver clusters calculated using the EAM and the *n*-body Gupta potential. Also the applied global optimization methods were different: BH for EAM and Aufbau/Abbau method for Gupta potential. The similarity function is based on the radial distances.

Another interesting issue is the comparison of the structures of Ag clusters obtained with different theoretical methods. Thus, the similarity functions for comparing clusters of Ag as calculated using the EAM and the *n*-body Gupta potential are shown in Figure 5. The results confirm the similarities of special sizes of high symmetry. For small (n<15) and many middle-sized clusters the two methods lead to similar structures, but for sizes n>80 the similarity decreases significantly and has a local minimum for n=83. It is interesting that neighboring structures of high-symmetry sizes, i.e., those with one or two atoms more/less, are often quite dissimilar according to the results of two different methods. This can be seen, e.g., for n=15,18,54,74, and 77.

### 3.2. Energetic Properties

Next, we discuss the energetic properties of Ag${}_{n}$ clusters. To this end, the so called stability function is useful. The stability function of a cluster with *n* atoms is defined as the second difference of energy with respect to size,

(8) $$\Delta^{2}E_{n}=E\left(n+1.1\right)+E\left(n-1.1\right)-2E\left(n.1\right).$$

**Figure 6:**
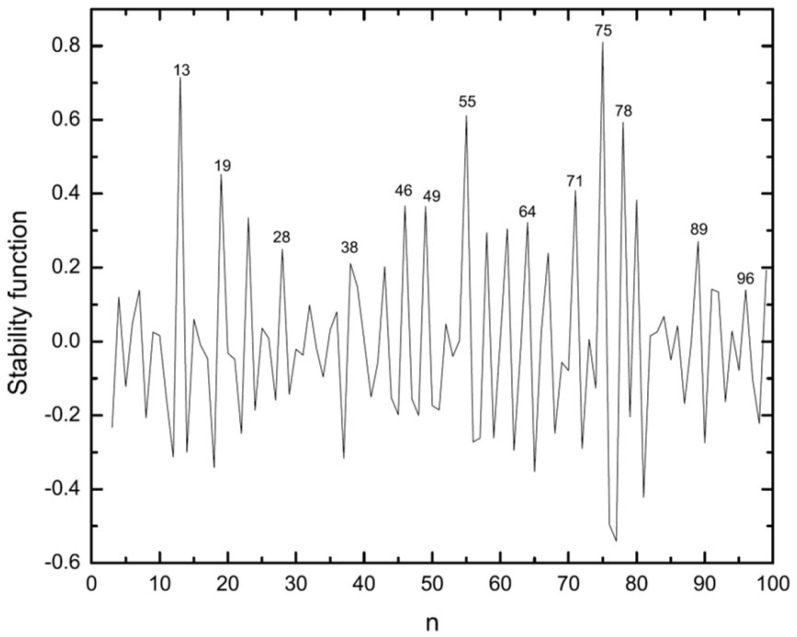
The stability function (in eV) of the silver clusters versus cluster size calculated using EAM-BH.

Here, E(n.k) is the energy of the $k^{th}$ lowest isomer of the Ag${}_{n}$ cluster. Plotting stability functions versus number of atoms, maxima will reveal the most stable clusters, i.e., magic numbers (Figure 6).

According to Figure 6, Ag${}_{75}$ is the most stable cluster in the studied size range and is followed by Ag${}_{55},$ Ag${}_{13}$, and Ag${}_{78}$. Although most of the magic clusters found in previous studies are recovered in our study there exist some exceptions. While Gupta and Sutton-Chen potentials find the Ag${}_{13}$ as the most stable one [15,16] EAM-BH identifies the Ag${}_{75}$ with a Marks decahedron structure ($D_{5h}$) as being more stable. On the other hand, in the present work, Ag${}_{78}$ with $C_{s}$ symmetry is found to be magic, instead of Ag${}_{79}$. These differences in stability functions are caused by differences in the model potentials.

For the range 80≤n≤100 Figure 6 shows that Ag${}_{80}$, Ag${}_{89}$, Ag${}_{96}$, and Ag${}_{99}$ are magic clusters. Additionally, the clusters for n=84,86, and 94 are more stable than their neighboring clusters, while n=91 appears to be almost as stable as n=92.

**Figure 7:**
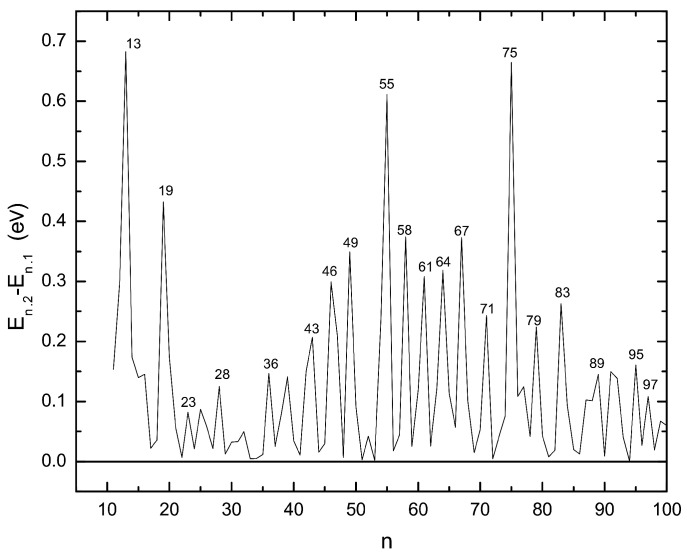
The difference between energies of energetically first and second lowest lying isomers of Ag clusters versus the number of atoms in the cluster as found in the EAM calculations.

The second quantity which can be used to identify stable clusters is the difference in the energies of the energetically lowest and second-lowest isomers (Figure 7). From this quantity one can also see how close in energy neighboring isomers are. As can be seen in Figure 7, Ag${}_{75}$ is again one of the stable clusters but in this case with a slightly lower stability than the Ag${}_{13}$ cluster which appears as the most stable one according to this criterion. Almost all of the stable clusters identified through the stability function (Figure 6) are recovered here but some exceptions exist. A noticeable difference is seen for Ag${}_{38}$ which, with a truncated octahedral structure, belongs to the stable clusters when considering the stability function, but the difference in energy of the first and second isomers is only small. In the n≥78 range, Ag${}_{79}$, Ag${}_{83}$, Ag${}_{95}$, and Ag${}_{97}$ appear as being more stable than other cluster sizes in their vicinity. All of these structures are based on the decahedral Ag${}_{75}$. Again the Ag${}_{91}$ and Ag${}_{92}$ clusters are very close to each other in stability. Comparing the results of the EAM calculations to the *n*-body Gupta results [16] we see the most subtle difference to occur for Ag${}_{79}$. This size shows a significant peak for the isomer energy difference in the *n*-body Gupta study, which is in contrast to our present result. The other difference is observed for Ag${}_{28}$ which we find to be a stable cluster while it exhibits no peak in the n−body Gupta results. The situation is reversed for Ag${}_{38}$ and Ag${}_{39}$.

Finally, the small differences in energy between the energetically lowest and the second-lowest isomer can explain why the energetic ordering in the EAM and the DFT calculations for some cluster sizes differs. Only very small differences are needed to change the relative order.

### 3.3. Electronic Properties

The electronic properties are accessible only through the DFT calculations. We shall here focus on the single isomer that is most stable for a given clusters size according to the DFT study. An isolated Ag atom possesses a filled *d* shell and a single *s* electron. Both types of orbitals and their interactions are important for the properties of the clusters, as we will demonstrate.

**Figure 8:**
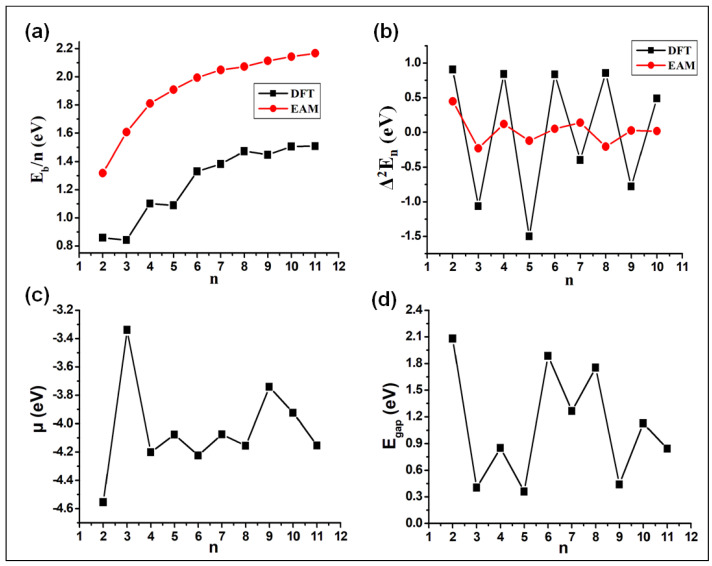
The variation of (**a**) $E_{b}$/*n*, (**b**) $\Delta^{2}E_{n}$, (**c**) μ, and (**d**) $E_{\mathrm{gap}}$ as a function of cluster size (*n*) as found in the DFT calculations and considering solely the energetically most stable isomer of these calculations. In (**a**,**b**) we also show the equivalent results from the EAM calculations (the red curves).

The binding energy per atom ($E_{b}$/*n*) of the Ag clusters is defined as

(9) $$E_{b}/n=\frac{nE_{\mathrm{Ag}}-E_{{\mathrm{Ag}}_{n}}}{n}$$

where E${}_{\mathrm{Ag}}$ and E${}_{{\mathrm{Ag}}_{n}}$ are the energies of a single Ag atom and the Ag${}_{n}$ cluster, respectively, and *n* is the cluster size. The variation of E${}_{b}$/*n* as a function of cluster size is shown in Figure 8a. One can observe a general trend of increasing stability with increasing size for the Ag clusters, a trend that often is found for clusters. However, the increase in stability is not smooth and exhibits a clear even–odd oscillation that can be explained through the odd number of electrons per atom [8]. The binding energy per atom is compared with the similar results from the EAM calculations. Besides being a more smooth function of cluster size, the EAM results are in general larger than the DFT ones.

The stability function is shown in Figure 8b against cluster size. The clusters with the most stable sizes (number of atoms) can be identified from the peaks in $\Delta^{2}E_{n}$ and are seen to correspond to clusters with an even number of atoms. This is in marked contrast to the results of the EAM calculations and demonstrate that for this property electronic effects are important. This trend can be directly related to the electron pairing effect, which strongly influences all the cluster properties. Similar trends have been observed in previous studies, too [55].

The vertical ionization energy (VIE) and vertical electron affinity (VEA) are calculated as the difference in the total energies between the ground states of the singly charged cation and the neutral species (VIE) as well as of the neutral species and the singly charged anion (VEA) all calculated for the optimized structure of the neutral species. Thus, electronic, but not structural, relaxation effects are included. The differences between those quantities and the negative energies of the HOMO (Highest Occupied Molecular Orbital) and of the LUMO (Lowest Unoccupied Molecular Orbital) are accordingly due to electronic relaxation effects. The calculated values of VIE and VEA are listed in Table 3. For clusters with more than just 3 atoms, there is a clear oscillatory behavior of both VIE and VEA with, moreover, VIE being low when VEA is large and vice versa. Also this behavior can be related to the even-odd oscillations in the total electron counts.

From the values of the VIE and VEA the chemical potential (μ) can be calculated,

(10) $$\mu =-\frac{1}{2}\left[\mathrm{VIE}+\mathrm{VEA}\right]$$

μ quantifies the escaping tendency of an electron, i.e., a higher value of μ value implies an increased reactivity. Figure 8c shows the variation of μ with respect to cluster size. The clusters with odd numbers have a higher value of μ, again a manifestation of the odd-even oscillatory behavior of the electronic properties of these clusters.

Further, employing the same level of theory, the energies of the HOMO and the LUMO, $E_{\mathrm{HOMO}}$ and $E_{\mathrm{LUMO}}$, and the band gap ($E_{\mathrm{gap}}=E_{\mathrm{LUMO}}-E_{\mathrm{HOMO}}$) were calculated and are listed in Table 3. By comparing the HOMO and LUMO orbital energies with the VIE and VEA values, respectively, significant differences are observed, indicating that electronic relaxation effects are important.

Generally, a smaller value of $E_{\mathrm{gap}}$ implies a lower energy required to excite the electrons from the valence to the conduction band, which corresponds to a higher chemical activity of the system. The computed $E_{\mathrm{gap}}$ values of the Ag${}_{n}$ clusters are plotted in Figure 8d as a function of cluster size. We observe that a low value of μ, seen for the even numbered cluster sizes, corresponds to a higher value of $E_{\mathrm{gap}}$, another indicator for the larger stability of those clusters. The Ag${}_{n}$ clusters with an even number of electrons have a closed shell and are hence more stable than the ones with an odd number of electrons. One can also recognize a larger variation and an overall decrease in the $E_{\mathrm{gap}}$ values of the clusters with even number of atoms as these clusters grow in size, an observation that also has been seen for other cluster systems [56,57].

Some comments on the magic-numbered Ag clusters are in place here. Magic-numbered clusters are characterized by a particularly high stability which can have more reasons. At first, packing (geometrical) effects can be important so that clusters with a high symmetry and hardly any low-coordinated atoms can be magic. These effects are included in the EAM approach. However, this is not the case for electronic effects that also can be responsible for particularly high stability. Thus, a low electron affinity and a high ionization potential (i.e., large $E_{\mathrm{gap}}$) can explain an enhanced stability [6]. In our DFT calculations we found that Ag${}_{6}$ and Ag${}_{8}$ can be classified as being magic, since they have a relatively high value of $E_{b}$/*n* along with higher VIE of 7.01 and 6.80 eV, respectively, and lower VEA of 1.44 and 1.51 eV, respectively. Moreover, $E_{\mathrm{gap}}$ for these clusters is relatively large, i.e., 1.89 and 1.75 eV, respectively. Therefore, Ag${}_{6}$ and Ag${}_{8}$ clusters are electronically the most stable clusters among all the clusters studied here. For Ag${}_{2}$, though the ionization potential is the highest (8.02 eV) and the electron affinity is the second lowest (0.56 eV) among the clusters studied here, $E_{b}$/*n* is low, implying that Ag${}_{2}$ cannot be considered as being a magic-number cluster.

A more detailed description of the electronic properties, in particular of catalytic properties, can be obtained from local descriptors or their atom-resolved analogues. The Fukui functions, $f^{+}\left(\vec{r}\right)$ and $f^{-}\left(\vec{r}\right)$, describe where in space electrons are added or removed when their number is changed. From these functions, atom-resolved, so-called condensed Fukui functions can be determined as suggested by Yang et al. [58]. $f_{k}^{+}$ and $f_{k}^{-}$, with *k* being an atom index, quantifies then the ability of a given atom in a molecule to react with nucleophilic and electrophilic reagents, respectively. The condensed Fukui functions are calculated according to

(11) $$\begin{array}{ccc} f_{k}^{+} & = & q_{k}\left(N+1\right)-q_{k}\left(N\right)\;\mathrm{for}\;\mathrm{nucleophilic}\;\mathrm{attack}\\ f_{k}^{-} & = & q_{k}\left(N\right)-q_{k}\left(N-1\right)\;\mathrm{for}\;\mathrm{electrophilic}\;\mathrm{attack} \end{array}$$

where $q_{k}\left(N\right)$, $q_{k}\left(N+1\right)$, and $q_{k}\left(N-1\right)$ present the charges of the *k*th atom in a cluster with *N*, N+1, and N−1 electrons, respectively.

The dual descriptor, $\Delta f\left(\vec{r}\right)=f^{+}\left(\vec{r}\right)-f^{-}\left(\vec{r}\right)$, is another useful descriptor for chemical activity of a system. Also from this, atom-resolved condensed functions can be introduced [59]. Formally, the definition of the $\Delta f_{k}$ is closely related to the condensed Fukui functions,

(12) $$\begin{array}{ccc} \Delta f_{k} & = & f_{k}^{+}-f_{k}^{-}=\left(q_{k}\left(N+1\right)-q_{k}\left(N\right)\right)-\left(q_{k}\left(N\right)-q_{k}\left(N-1\right)\right)\\ & = & q_{k}\left(N+1\right)-2q_{k}\left(N\right)+q_{k}\left(N-1\right). \end{array}$$

$\Delta f_{k}>0$ when the *k*th site favors a nucleophilic attack, whereas $\Delta f_{k}<0$ when the site favors an electrophilic attack.

**Figure 9:**
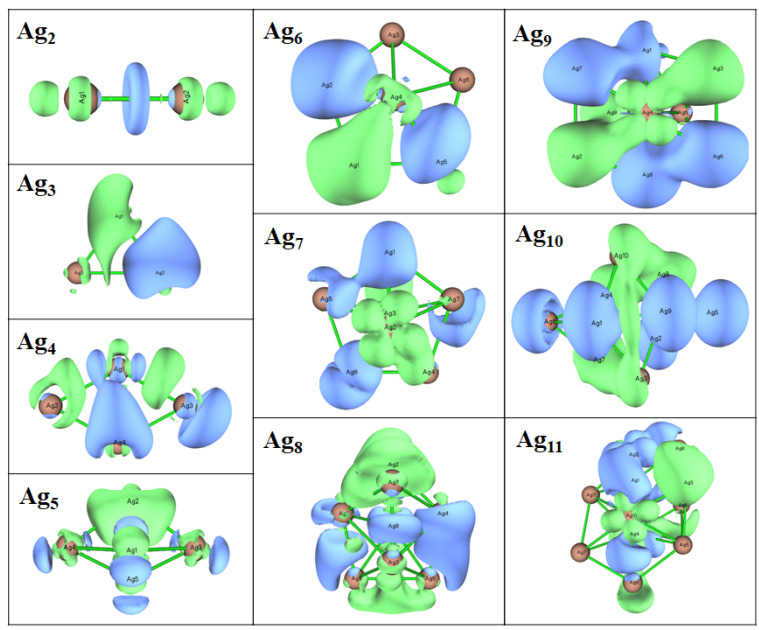
The dual descriptor ($\Delta f(\vec{r})$) of the most stable Ag${}_{n}$ (*n* = 2–11) clusters according to the DFT calculations. Green and blue regions correspond to positive and negative regions, respectively.

As discussed above, the Ag${}_{n}$ clusters tend to accept electrons and hence favor the attack of a nucleophile. In order to identify the most favorable sites for nucleophilic attack within a cluster unit, plots of $\Delta f(\vec{r})$ were generated using the Multiwfn program [60]. Figure 9 shows $\Delta f(\vec{r})$ with green and blue regions corresponding to positive and negative regions, respectively, that are favorable for nucleophilic and electrophilic attack, respectively. It is interesting that the function is not localized to certain atoms, for instance low-coordinated ones, but spread out over the complete cluster. We speculate that this could be a consequence of Ag being a metal.

**Figure 10:**
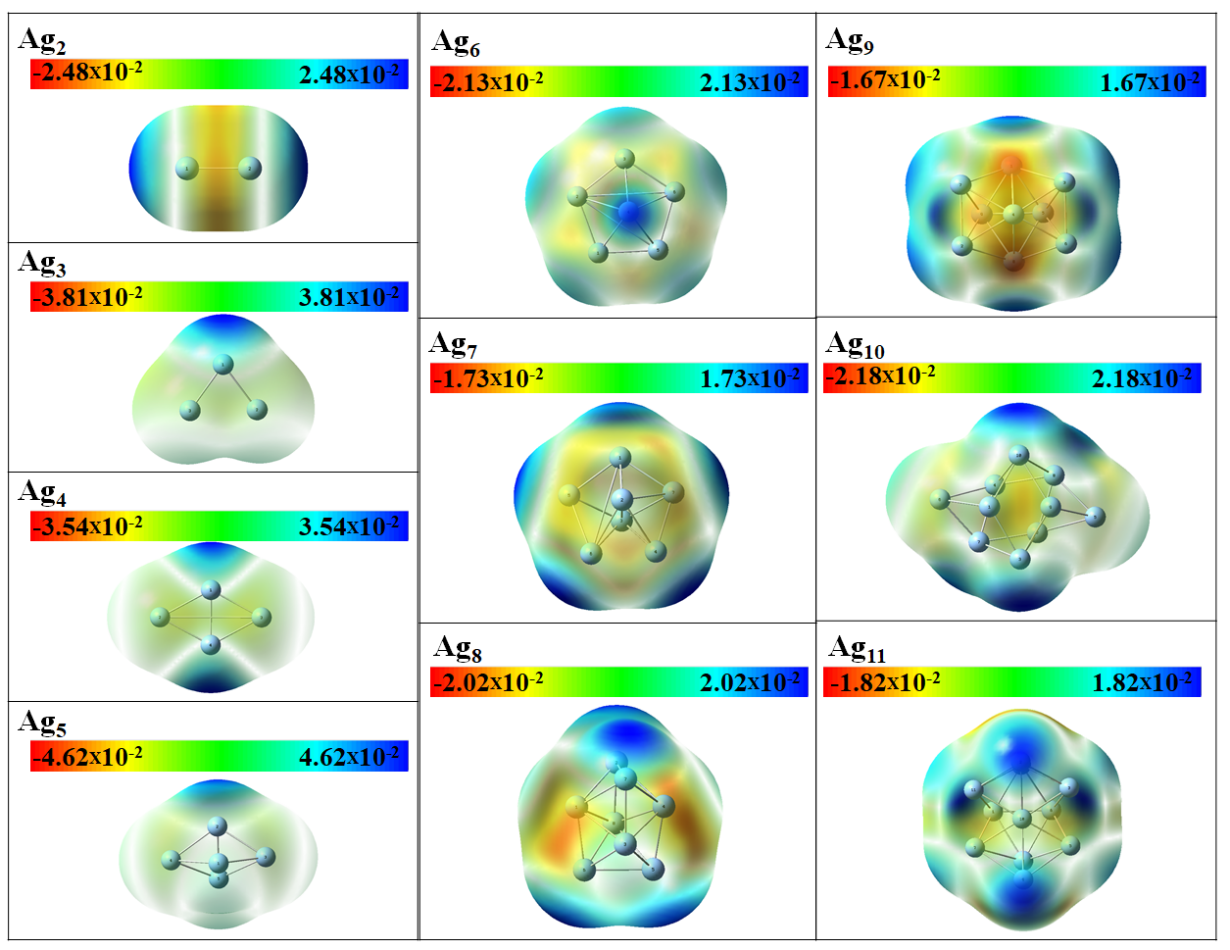
Molecular electrostatic (MEP) of the most stable Ag${}_{n}$ (*n* = 2–11) clusters according to the DFT calculations. The potential is shown on a surface of constant electron density (0.0004 a.u.) and the scales in each panel are given in a.u.

Finally, also the molecular electrostatic potential (MEP) shown in Figure 10 supports the concept that the electrons in the Ag${}_{n}$ clusters are delocalized over the complete system, as was also seen for $\Delta f(\vec{r})$.

### 3.4. Growth Patterns

For studying growth pattern, different approaches can be applied. For instance, one may compare the sequence of magic clusters to known sets of magic numbers for the possible growth patterns. An alternative that we shall pursue here is to make use of the concept of similarity functions.

There are two possible forms of icosahedral growth, i.e., MIC/Mackay or fcc covering and TIC/polyicosahedral, which have been proposed and used by various authors for different types of clusters [24,61]. Within the MIC/Mackay covering growth, new atoms are added to the top of the edges and vertices of the first icosahedron, formed at n=13. This leads to a Mackay icosahedral at n=55. Instead, according to the TIC/polyicosahedral growth mechanism, new atoms are added to the top of the lower-layer atoms at the center of faces (T sites) [61]. For MIC and TIC patterns, the sets of expected magic clusters differ.

Comparing our results for silver clusters (Figure 6 and Figure 7) with data for the MIC and TIC magic numbers suggests that they grow according to the TIC pattern from the icosahedron structure of n=13 up to n=26. Subsequently, the growth follows the MIC pattern, with an exception for n=38, till the second complete icosahedron for n=55 has been formed. From n=56 up to n=71 the sequence of magic clusters again coincide with the TIC growth model. For larger sizes (n≥71), magic clusters as identified with the help of the stability function do not agree with the TIC growth. Nevertheless, the stable clusters according to the first and second isomers energy difference coincide with those of the MIC pattern at n=71,83, and 92. It should be noticed that in the range *n* = 32–38, if one looks at the structures, they appear to follow a growth according to the fcc growth pattern while the magic clusters in this range are those proposed by MIC. This demonstrates the complexity of the growth especially in this size range.

As mentioned above, another tool to study the growth of silver clusters is provided by the similarity functions. With these we can compare each cluster size to the neighboring cluster with one atom less. Then, sudden changes in structures can be identified through this similarity function as a function of cluster size. To calculate the similarity function for clusters with *n* and *n* − 1 atoms, all the *n* possible cases of removing one atom from the *n*-atomic cluster is considered. Then, similarities of the *n* − 1 atomic cluster with each of these possible structures are computed according to Equation (5). Finally, the largest value of *S* is chosen as the value of the similarity function.

**Figure 11:**
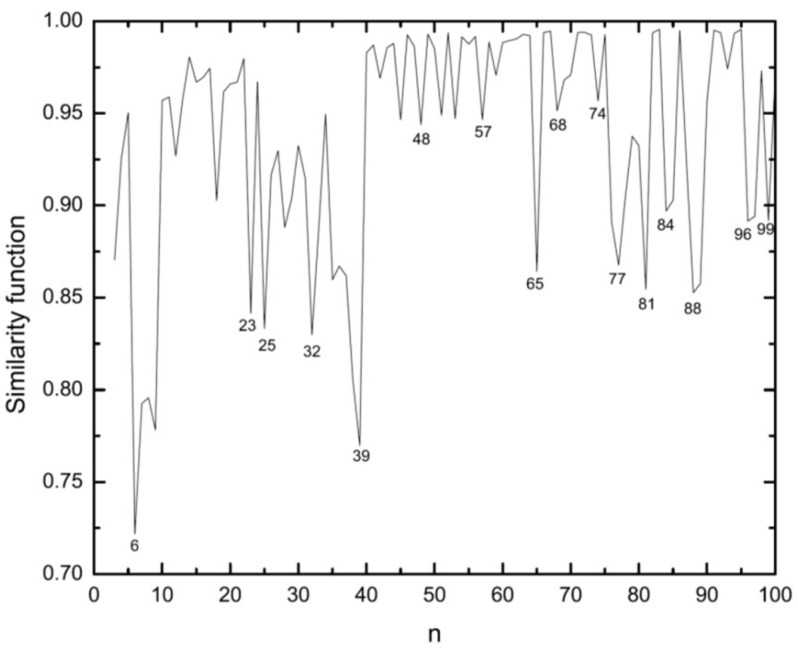
Similarity function of *n* atomic clusters when comparing with the clusters with *n* − 1 atoms. This can be used in identifying the structural changes due to a growth. The similarity function is based on the interatomic distances.

Figure 11 shows the results. Sudden changes, which correspond to irregular growth, happen frequently for smaller sizes up to n=39. In the range 39<n<65 the growth follows a more smooth pattern, but again for n≥65 the irregularity reappears even if not as pronounced as before. Despite two sharp structural changes at n=23 and 25, the growth follows an icosahedral pattern until n=32 where another sudden change occurs. This change in similarity function at n=25 can be identified as the change from TIC to MIC growth. As previously mentioned for 32≤n<39, by looking at the structures, an fcc growth can be observed which results in the truncated octahedron at n=38. From n=39 the layered icosahedral growth is initiated and the high-symmetry structure of the second Mackay icosahedron is formed at n=55.

A further parameter that can be helpful in identifying the growth pattern is the minimum coordination number. From this quantity one can see how new atoms are added to a cluster when constructing the larger cluster directly from the smaller one. Low values for the minimum coordination number (i.e., 3 or 4) indicate that new atoms are added to the surface of the lower-sized cluster whereas higher values as 5 or 6 are due to the addition of new atoms to the inner regions of the cluster.

**Figure 12:**
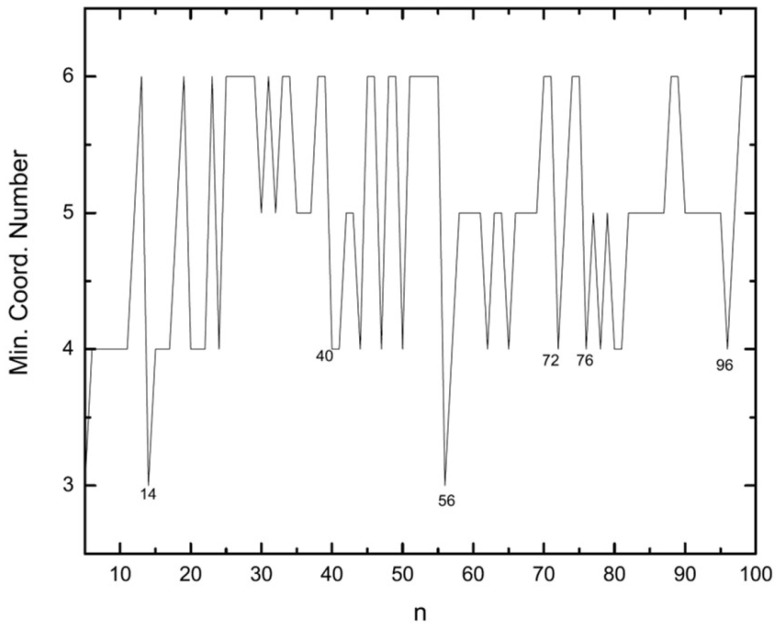
Minimum coordination number in each cluster size versus the number of atoms.

As can be seen in Figure 12, when starting with the symmetric structures, the growth continues by addition of atoms to the surface, a phenomenon which is more obvious for the special cases of icosahedra at n=13 and n=55 where the low coordination number of 3 happens for n=14 and 56. For larger sizes, n≥2, the growth takes mainly place by addition of atoms to the surface except at n=96. The surface growth can also be seen for 25≤n<40.

### 3.5. Vibrational Frequencies

**Figure 13:**
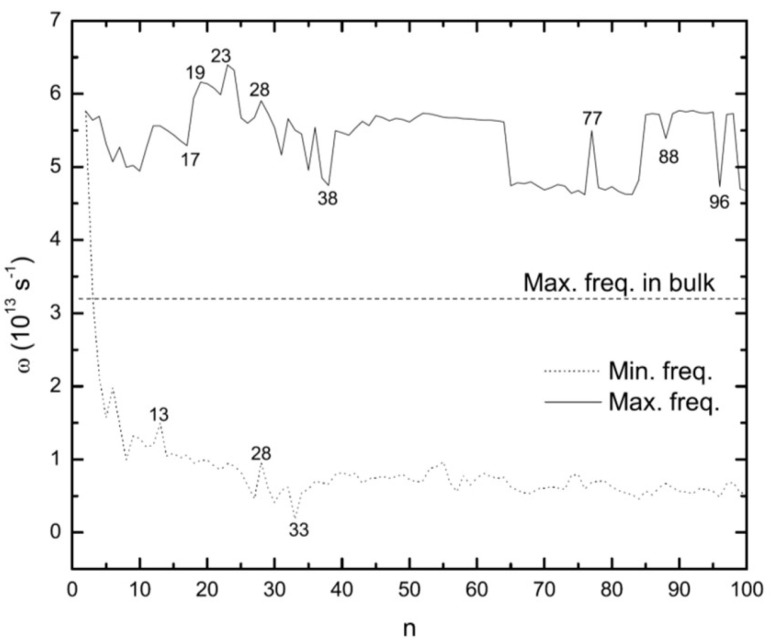
Maxima and minima of vibrational frequencies for the optimized Ag${}_{n}$ clusters. The dashed line shows the maximum value of the vibrational frequencies for bulk silver.

A more complete understanding of the properties of silver clusters requires knowledge about their vibrational frequencies. Here, we have used our results for the lowest-energy structures to determine their lowest and highest vibrational frequencies. For this aim the harmonic approximation has been utilized [62]. Again with the help of the EAM, the 3n×3n dynamical matrix of $D_{li,mj}=M^{-1}\times \frac{\partial^{2}E}{\partial R_{li}\partial R_{mj}}$ is calculated. $R_{li}$ is the *i*th cartesian coordinates (i=x,y,z) of atom *l*, and *M* is the atomic mass. Numerical diagonalization of this matrix gives the normal modes and the corresponding eigenvectors. The largest and smallest non-zero vibrational frequencies are shown in Figure 13.

For small sizes the large oscillations in frequencies can be interpreted as caused by the addition of a single atom and/or extreme structural changes between fcc and icosahedral motifs, whereas for larger clusters the oscillations are mainly due to structural changes. For fcc (Ag${}_{38}$) and decahedral (Ag${}_{75}$) structures, the largest frequencies are smaller than those of the icosahedral structures (Ag${}_{13}$, Ag${}_{19}$, Ag${}_{23}$, and Ag${}_{55}$). The values for fcc and decahedral structures are closer to the bulk maximum vibrational frequencies; cf. Figure 13.

Because of the high strains in icosahedral and polyicosahedral structures, their frequencies can be related to the existence of large restoring forces. On the contrary, for fcc and decahedral structures the strains are smaller. Instead, they are close to having values as in bulk. The highest values of the frequencies are seen for 18≤n≤28 (Figure 13). In this range, all clusters are based on polyicosahedral structures. Special cases of n=19 and n=23 are double and triple icosahedra, respectively. Interesting cases are the maximum frequencies of Ag${}_{77}$, Ag${}_{88}$, and Ag${}_{96}$. Interestingly, Ag${}_{77}$ has a high value of the maximum frequency in comparison to the neighboring decahedral structures. For Ag${}_{88}$ and Ag${}_{96}$ the case is completely reversed and they have lower frequencies in comparison to other clusters in their vicinity.

For the lowest vibrational frequencies the above discussion still applies. Again for the range 18≤n≤28 as well as for the icosahedral structure of Ag${}_{55}$ the lowest frequency takes higher values compared to other sizes. The lowest value is found for Ag${}_{33}$. This might be due to a sudden change in structural morphologies and growth pattern, i.e., from polyicosahedral to fcc.

Some few further conclusions can be drawn from our results on the vibrational frequencies. First, however, it is relevant to emphasize that even for a cubic cluster of 5×5×5=125 atoms (i.e., slightly larger than the largest clusters of the present study), only 3×3×3=27 (i.e., 22%) are not surface atoms, so that for all the clusters of the present study, surface effects will dominate those properties that are non-local like vibrational properties. This is one reason for the irregular behavior observed in Figure 13 and it also makes an identification of general trends for the vibrational properties hardly possible. On the other hand, the fact that all vibrational frequencies are real and positive demonstrates that all structures are local total-energy minima. Moreover, as demonstrated in our earlier studies on thermodynamic properties of clusters [63,64,65], the clusters for which the lowest vibrational frequencies take the lowest (highest) values are those for which the vibrational heat capacity per vibrational mode at not too elevated temperatures takes the largest (smallest) values. Such clusters are, accordingly, most (least) prone to absorb heat. This suggests that Ag${}_{33}$ is the cluster that will be easiest vibrationally excited.

## 4. Conclusions

In the present work we have presented results of a theoretical study devoted to determining energetical, electronic, and structural properties of three energetically low-lying isomers of silver clusters with *n* = 2–100 atoms. Thereby, we have used two different computational methods, i.e., the EAM and basin-hopping methods for the clusters with *n* = 2–100 atoms, whereas for the smaller systems, *n* = 2–11, we used a much more accurate DFT method. The simplified description of the interatomic interactions as implemented in the EAM was, of course, connected with the disadvantage that occasionally slightly inaccurate results could be obtained, as could be seen when comparing with the DFT results, but possessed the advantage that a large class of systems could be treated, as we have demonstrated here. Subsequently, by using different descriptors specifically designed for the task at hand we obtained a more detailed understanding of the energetic and structural properties of the clusters and could compare various systems directly. From the DFT results a detailed understanding of the importance of electronic effects could be obtained. Indeed, many features, like the even-odd oscillatory behavior of certain descriptors, could be captured only through the DFT results.

For the simplified approaches, we studied the effects of using different potential models and/or different global optimization algorithms by comparing properties of the obtained structures with those of previous studies. The growth process of the silver clusters was found to follow the icosahedral growth pattern for the largest parts of the size range of our study, but with some islands of fcc growth. Nevertheless, for larger sizes the growth was more complicated. Most of the magic clusters, found previously in other studies, were recovered here. In the range of n>80 some highly stable structures were obtained for the first time. In this range, according to the stability function, sizes of n=78,80,89, and 96 are more stable than others. In addition, based on the energy differences of the first and second lowest energy isomers, n=79,83,89,95, and 97 are magic sizes.

Vibrational frequencies of the global minima structures of silver clusters were also calculated, whereby we here focused on the smallest and largest values. As expected, icosahedral structures have higher values compared to fcc and decahedral ones. A high value for the highest vibrational frequence was observed also for n=77, although its structure is not that of an icosahedron.

As demonstrated in our earlier studies on other metal clusters [63,64,65] the results obtained in the present study allow also for a detailed discussion of the thermodynamic properties of the clusters. This can be obtained by using the energetics, structures, symmetry properties, and vibrational properties of more (in our case, three) isomers of the clusters. From this information, a temperature-dependent stability function based on the Gibbs free energy can be determined, allowing for studying the temperature dependence of the relative stability of the clusters, both as a function of size and as a function of energetic ordering at T=0. To do this here is, however, beyond the scope of the present study.

Interesting was it that even the smallest Ag${}_{n}$ clusters of the present study showed signatures that we interpret as manifestations of a metallic behavior. Thus, the Fukui functions were delocalized over the complete system and also the electrostatic potential outside the cluster was fairly unstructured.

## Figures and Tables

**Table 1:** Point groups of the optimized Ag${}_{n}$ clusters as obtained with the DFT calculations. n.1, n.2, and n.3 represents the results for the energetically lowest, 2nd lowest, and 3rd lowest isomer, respectively, from the EAM—and not the DFT—calculations.

*n*	n.1	n.2	n.3
2	$D_{\infty h}$		
3	$C_{2v}$		
4	$D_{2h}$		
5	$C_{2v}$		
6	$C_{5v}$	$C_{5v}$	
7	$D_{5h}$	$C_{3v}$	$D_{5h}$
8	$D_{2d}$	$C_{s}$	$C_{2v}$
9	$C_{2v}$	$C_{1}$	$C_{s}$
10	$C_{1}$	$C_{2}$	$C_{2v}$
11	$C_{2v}$	$C_{2v}$	$C_{2v}$

**Table 2:** Point groups of the optimized Ag${}_{n}$ clusters from the EAM calculations. n.1, n.2, and n.3 represents the results for the energetically lowest, 2nd lowest, and 3rd lowest isomer, respectively.

*n*	n.1	n.2	n.3	*n*	n.1	n.2	n.3	*n*	n.1	n.2	n.3
2	$D_{\infty h}$			35	$C_{s}$	$D_{3}$	$C_{2v}$	68	$C_{1}$	$C_{1}$	$C_{s}$
3	$D_{3h}$			36	$C_{s}$	$C_{1}$	$C_{2}$	69	$C_{1}$	$C_{1}$	$C_{1}$
4	$T_{d}$			37	$C_{s}$	$C_{3v}$	$C_{2}$	70	$C_{s}$	$C_{1}$	$C_{1}$
5	$D_{3h}$			38	$O_{h}$	$C_{5v}$	$C_{s}$	71	$C_{2v}$	$C_{5}$	$C_{5v}$
6	$O_{h}$	$C_{2v}$		39	$C_{5v}$	$C_{5}$	$C_{4v}$	72	$C_{s}$	$C_{1}$	$C_{1}$
7	$D_{5h}$	$C_{3v}$	$C_{2}$	40	$C_{s}$	$C_{s}$	$C_{1}$	73	$C_{2v}$	$C_{s}$	$C_{S}$
8	$D_{2d}$	$C_{s}$	$D_{3d}$	41	$C_{s}$	$C_{s}$	$C_{1}$	74	$C_{5v}$	$C_{1}$	$C_{s}$
9	$C_{2v}$	$D_{3h}$	$C_{2v}$	42	$C_{s}$	$C_{1}$	$C_{2v}$	75	$D_{5h}$	$C_{s}$	$C_{s}$
10	$C_{3v}$	$C_{2}$	$C_{2v}$	43	$C_{s}$	$C_{s}$	$C_{1}$	76	$C_{1}$	$C_{s}$	$C_{1}$
11	$C_{2v}$	$C_{2}$	$C_{2}$	44	$C_{1}$	$C_{1}$	$C_{s}$	77	$C_{s}$	$C_{1}$	$C_{2v}$
12	$C_{5v}$	$D_{2d}$	$C_{1}$	45	$C_{s}$	$C_{1}$	$C_{1}$	78	$C_{s}$	$C_{1}$	$C_{s}$
13	$I_{h}$	$C_{s}$	$C_{s}$	46	$C_{2v}$	$C_{s}$	$C_{1}$	79	$C_{s}$	$C_{1}$	$C_{1}$
14	$C_{3v}$	$C_{2v}$	$C_{1}$	47	$C_{1}$	$C_{1}$	$C_{1}$	80	$C_{s}$	$C_{1}$	$C_{s}$
15	$C_{2v}$	$D_{6d}$	$C_{2v}$	48	$C_{s}$	$C_{s}$	$C_{1}$	81	$C_{1}$	$C_{s}$	$C_{1}$
16	$C_{s}$	$C_{s}$	$C_{1}$	49	$C_{3v}$	$C_{s}$	$C_{s}$	82	$C_{1}$	$C_{s}$	$C_{s}$
17	$C_{2}$	$C_{s}$	$C_{s}$	50	$C_{s}$	$C_{s}$	$C_{s}$	83	$C_{s}$	$C_{1}$	$C_{1}$
18	$C_{s}$	$C_{5v}$	$C_{s}$	51	$C_{s}$	$C_{s}$	$C_{1}$	84	$C_{s}$	$C_{1}$	$C_{s}$
19	$D_{5h}$	$C_{1}$	$C_{s}$	52	$C_{2v}$	$C_{3v}$	$C_{s}$	85	$C_{1}$	$C_{1}$	$C_{1}$
20	$C_{2v}$	$C_{s}$	$D_{3d}$	53	$C_{2v}$	$D_{5d}$	$C_{2v}$	86	$C_{s}$	$C_{1}$	$C_{1}$
21	$C_{1}$	$C_{2v}$	$C_{s}$	54	$C_{5v}$	$I_{h}$	$C_{2v}$	87	$C_{s}$	$C_{1}$	$C_{2}$
22	$C_{1}$	$C_{s}$	$C_{s}$	55	$I_{h}$	$C_{s}$	$C_{1}$	88	$C_{s}$	$C_{1}$	$C_{1}$
23	$D_{3h}$	$D_{3h}$	$C_{2}$	56	$C_{3v}$	$C_{s}$	$C_{s}$	89	$C_{3v}$	$C_{s}$	$C_{1}$
24	$C_{2v}$	$C_{s}$	$D_{3}$	57	$C_{s}$	$C_{s}$	$C_{s}$	90	$C_{s}$	$C_{1}$	$C_{1}$
25	$C_{3}$	$C_{s}$	$C_{1}$	58	$C_{3v}$	$C_{s}$	$C_{1}$	91	$C_{s}$	$C_{s}$	$C_{1}$
26	$C_{1}$	$T_{d}$	$C_{2v}$	59	$C_{2v}$	$C_{1}$	$C_{1}$	92	$C_{3v}$	$C_{1}$	$C_{1}$
27	$C_{s}$	$C_{s}$	$C_{2}$	60	$C_{s}$	$C_{s}$	$C_{s}$	93	$C_{1}$	$C_{1}$	$C_{1}$
28	*T*	$C_{1}$	$C_{3v}$	61	$C_{2v}$	$C_{1}$	$C_{1}$	94	$C_{1}$	$C_{1}$	$C_{1}$
29	$C_{3}$	$C_{2v}$	$C_{2}$	62	$C_{1}$	$C_{1}$	$C_{1}$	95	$C_{1}$	$C_{1}$	$C_{1}$
30	$C_{s}$	$C_{2v}$	$C_{1}$	63	$C_{1}$	$C_{1}$	$C_{s}$	96	$C_{1}$	$C_{1}$	$C_{s}$
31	$C_{3}$	$C_{2v}$	$C_{s}$	64	$C_{s}$	$C_{1}$	$C_{1}$	97	$C_{1}$	$C_{1}$	$C_{1}$
32	$C_{2v}$	$D_{3}$	$C_{1}$	65	$C_{2v}$	$C_{1}$	$C_{s}$	98	$C_{s}$	$C_{1}$	$C_{1}$
33	$C_{2}$	$C_{s}$	$C_{s}$	66	$C_{s}$	$C_{1}$	$C_{1}$	99	$C_{s}$	$C_{2v}$	$C_{1}$
34	$C_{s}$	$C_{s}$	$C_{s}$	67	$C_{2v}$	$C_{s}$	$C_{s}$	100	$C_{5v}$	$C_{1}$	$C_{s}$

**Table 3:** Calculated values of VIE, VEA, μ, the energy of HOMO, that of LUMO, and E${}_{\mathrm{gap}}$ of Ag${}_{n}$ (*n* = 2–11) clusters. All quantities are given in eV.

Cluster Size	VIE	VEA	μ	$E_{\mathrm{HOMO}}$	$E_{\mathrm{LUMO}}$	$E_{\mathrm{gap}}$
2	8.02	1.08	−4.55	−5.26	−3.18	2.08
3	6.11	0.56	−3.34	−3.90	−3.49	0.40
4	6.67	1.73	−4.20	−4.51	−3.66	0.85
5	6.30	1.86	−4.08	−4.23	−3.87	0.36
6	7.01	1.44	−4.23	−5.01	−3.13	1.89
7	6.15	2.00	−4.08	−4.22	−2.95	1.26
8	6.80	1.51	−4.16	−4.90	−3.14	1.75
9	5.57	1.91	−3.74	−3.86	−3.42	0.44
10	6.13	1.72	−3.93	−4.41	−3.29	1.13
11	5.92	2.38	−4.15	−4.26	−3.42	0.84

## Data Availability

All data can be obtained from the authors upon request.
